# Hydrophobic interaction chromatography of proteins: Studies of unfolding upon adsorption by isothermal titration calorimetry

**DOI:** 10.1002/jssc.201800016

**Published:** 2018-06-26

**Authors:** Agnes Rodler, Beate Beyer, Rene Ueberbacher, Rainer Hahn, Alois Jungbauer

**Affiliations:** ^1^ Department of Biotechnology University of Natural Resources and Life Sciences Vienna Vienna Austria; ^2^ Austrian Centre of Industrial Biotechnology Vienna Austria

**Keywords:** bovine serum albumin, lactoglobulin, stationary phases, van't Hoff analysis

## Abstract

Heat of adsorption is an excellent measure for adsorption strength and, therefore, very useful to study the influence of salt and temperature in hydrophobic interaction chromatography. The adsorption of bovine serum albumin and β‐lactoglobulin to Toyopearl Butyl‐650 M was studied with isothermal titration calorimetry to follow the unfolding of proteins on hydrophobic surfaces. Isothermal titration calorimetry is established as an experimental method to track conformational changes of proteins on stationary phases. Experiments were carried out at two different salt concentrations and five different temperatures. Protein unfolding, as indicated by large changes of molar enthalpy of adsorption Δ*h*_ads_, was observed to be dependent on temperature and salt concentration. Δ*h*_ads_ were significantly higher for bovine serum albumin and ranged from 578 (288 K) to 811 (308 K) kJ/mol for 1.2 mol/kg ammonium sulfate. Δ*h*_ads_ for β‐lactoglobulin ranged from 129 kJ/mol (288 K) to 186 kJ/mol (308 K). For both proteins, Δ*h*_ads_ increased with increasing temperature. The influence of salt concentration on Δ*h*_ads_ was also more pronounced for bovine serum albumin than for β‐lactoglobulin. The comparison of retention analysis evaluated by the van't Hoff algorithm shows that beyond adsorption other processes occur simultaneously. Further interpretation such as unfolding upon adsorption needs other in situ techniques.

**Article Related Abbreviations**
ATR: attenuated total reflectance
ITC: isothermal titration calorimetry

## 1 INTRODUCTION

Hydrophobic stationary phases can induce conformational changes of proteins adsorbed to the surface 1. The extent of these changes is governed by factors such as salt concentration and temperature. The occurrence of conformational changes can be a drawback, especially for the purification of proteins on a preparative scale 2, 3, 4, 5. This requires an in‐depth characterization of chromatographic media under operating conditions 6. Various methods such as pulse response experiments, attenuated total reflectance (ATR) FTIR spectroscopy 7, hydrogen–deuterium exchange as analyzed by MS 8, 9, circular dichroism and Raman spectroscopy as well as isothermal titration calorimetry (ITC) have been used to investigate conformational changes. Kinetics for this so called “protein spreading” has been determined for the adsorption of BSA and β‐lactoglobulin to Toyopearl Butyl‐650 M 10. Very recently, nano differential scanning fluorimetry has been employed to characterize stability of proteins in the adsorbed state 11.

In the last decade, ITC has become an essential method to investigate structure and interaction mechanisms in the adsorbed state 12, 13, 14, 15, 16. Chen and his group have established a method to measure thermodynamic quantities of proteins adsorbed to HIC sorbents with ITC 17, 18, 19, 20. They studied the adsorption of various proteins to Butyl‐ and Octyl‐Sepharose and to butyl‐ and phenyl‐derivatized Toyopearl 650 M resins. Overviews about the large spectrum of ITC applications, which have been extensively used for studying protein–protein and protein–peptide interactions, are given in 21, 22, 23.

Recent investigations with ITC corroborated earlier studies conducted with pulse response experiments and ATR FTIR spectroscopy with respect to protein unfolding upon adsorption onto a hydrophobic surface 2, 7. There were discrepancies in the values of the specific enthalpy of adsorption (Δ*h*_ads_) determined from van´t Hoff analysis and from calorimetric measurements 24, 25. Discrepancies between Δ*h*_ads_ *vH* and Δ*h*_ads_ *ITC* with respect to the adsorption of proteins to a hydrophobic surface were attributed to the fact that van´t Hoff plots are based on a reversible equilibrium process whereas ITC measures the sum of a variety of enthalpy changes including protein adsorption, dilution effects, and conformational changes 20.

We obtained the thermodynamic parameters using two different commercially available calorimeters at various temperatures. This approach was chosen because there is generally a lack of replicate data in the determination of adsorption as in many cases, data from single measurements are presented. Additionally, contrary to liquid/liquid systems, no standard test reaction for solid adsorption has been established.

A recent publication proposed that the discrepancies between calorimetrically determined Δ*h*_ads_ values and those derived from nonlinear van´t Hoff plots arise from conformational changes of the protein induced by binding to the hydrophobic surface 26. In an extension of these observations, we have investigated the adsorption of two model proteins, BSA and β‐lactoglobulin, to Toyopearl Butyl‐650 M, a material where conformational changes cannot be detected by ATR FTIR spectroscopy due to the lack of translucency of its base matrix. Here, calorimetric investigations are employed to circumvent this drawback for the characterization of polymethacrylate‐based media. Isothermal titration calorimetry measurements of the adsorption to Toyopearl Butyl‐650 M are correlated with the results of unfolding data determined by pulse response experiments to extract information about possible conformational changes.

Both BSA and β‐lactoglobulin are classical model proteins and both their adsorption behavior and their tendency to unfold in HIC is well investigated 7. These proteins do not have the huge economic impact for the biotech industry that antibodies for example have. Yet for a fundamental research study like ours, it is more appropriate to use model proteins, for which a lot of data is available in the literature to be able to put the obtained results into context with previous findings.

While BSA is a relatively big serum protein having mainly alpha helical structure, with a molecular weight of 66 kDa which tends to form larger aggregates, β‐lactoglobulin, the major whey protein in cow and sheep milk, has predominantly a β‐sheet secondary structure and a molecular weight of only 18 kDa. It is present mainly in its dimeric form at neutral pH. In terms of the adiabatic compressibility and their behavior in HIC, BSA is referred to as a “soft” protein having a higher tendency to unfold upon adsorption in HIC than β‐lactoglobulin, which has lower compressibility and is thus considered more “rigid” than BSA 27. Based on this knowledge, it was of interest to investigate in more detail, if and how pronounced this behavior would manifest itself in terms of energies.

It was also an additional goal to monitor a total set of experiments to see if an influence of a chemically different base matrix (methacrylate‐ versus agarose‐based polymer) could be detected.

## 2 MATERIALS AND METHODS

### 2.1 Instrumentation

For batch adsorption experiments, the Stuart SB3 tube rotator from Barloworld Scientific (Staffordshire, UK) was used. Batch adsorption experiments were performed at a defined temperature in the 2023 Minicoldlab from LKB Bromma (Sweden). For ITC experiments, the Thermal Activity Monitor (TAM) III nanocalorimeter from Thermometric AB (Jarfälla, Sweden), and the VP‐ITC calorimeter from MicroCal (Northampton, USA) were used. Before the measurements, samples were degassed in the Thermovac from MicroCal (Northampton, USA).

### 2.2 Stationary phases

Butyl Sepharose 4 Fast Flow (FF) was purchased from GE Healthcare (Uppsala, Sweden), Toyopearl Butyl‐650 M was acquired from Tosoh Bioscience (Stuttgart, Germany). For blank experiments, underivatized stationary phases were used: Sepharose 4 Fast Flow was available from Pharmacia (Uppsala, Sweden), Toyopearl without ligand (Toyopearl HW‐65) was purchased from Tosoh Bioscience (Stuttgart, Germany).

### 2.3 Buffers and proteins

(NH_4_)2SO_4_ at different molalities (0.7 and 1.2 mol/kg) was added to a 20 mM sodium phosphate buffer. A pH of 7.3 was used. All buffers were filtered through 0.22 μm filters manufactured by Millipore (Bedford, USA). The reagents were acquired from Merck (Vienna, Austria) and were of analytical grade. BSA (A6003; essentially fatty acid free) and β‐lactoglobulin (L3908) were purchased from Sigma–Aldrich (Vienna, Austria). For calorimeter standard tests, BaCl_2_·2H_2_O was purchased from Merck (Vienna, Austria) and 18‐crown‐6 ether was acquired from Fluka (Buchs, Switzerland).

### 2.4 Batch adsorption experiments

Batch adsorption experiments were used to determine equilibrium adsorption isotherms. Protein solutions of different concentrations and specified amounts of stationary phase slurry with a starting resin concentration of 50% v/v were incubated together in 1.5 mL tubes under constant mixing for 3 h at 14 rpm. After 15 min settling time, the supernatant was filtered through a 0.22 μm filter. Residual protein concentration in the supernatant was measured at 280 nm with a Varian Cary50 UV/VIS spectrophotometer (Palo Alto, USA). The amount of bound protein was determined by mass balance.

### 2.5 ITC measurements

The TAM III nanocalorimeter was equilibrated for 24–48 h at each measuring temperature to achieve a stable baseline. Before each experiment, the TAM III was calibrated with a dynamic calibration. Glass ampoules (1 mL) held each sample and a gold propeller turning at 120 rpm was used for stirring. Proteins were injected with a Hamilton syringe (250 μL) fitted with a stainless‐steel cannula. The stationary phase was suspended in buffer (1:4), and 800 μL of slurry was placed into the sample and reference ampoules. Additional buffer (50 μL) was added to the reference ampoule. The injection syringe was filled with protein solution (approximately 6 mg/mL). Exact protein concentration was determined by UV spectroscopy at 280 nm. Stationary phase slurry and protein solutions were carefully degassed; the slurries were not allowed to stir to avoid grinding of the particles.

For the VP‐ITC calorimeter, the working volume of the cells was 1.42 mL. The sample cell was filled with resin slurry (1:4) and the same volume of degassed HPLC grade water was added to the reference cell.

Protein solution was drawn into a 250 μL syringe, and titrations were conducted by the addition of 10 μL aliquots to the suspended stationary phase in the sample cell under a stirring rate of 502 rpm (using Toyopearl Butyl‐650 M and Toyopearl HW‐65) and 307 rpm (for Butyl Sepharose 4 FF and Sepharose Fast Flow). The higher stirring rate for Toyopearl Butyl‐650 M was chosen to avoid settling of the resin particles. The reference power was 5 μcal/s with a filter period of 2 s. Consecutive injections of 10 μL were used for both calorimeters. For a protein concentration of 6 mg/mL, one injection of 10 μL corresponded to an average amount of 1 nmol BSA and 3.3 nmol β‐lactoglobulin.

Time spacing between the injections was adjusted to allow the signal to return to the baseline. Between experiments, the system was thoroughly cleaned with detergent (Decon90), ethanol, and HPLC‐grade water. For evaluation of the performance of the calorimeters, the titration of BaCl_2_·2H_2_O to 18‐crown‐6 ether was carried out on both calorimeters according to Caldwell and Yan 28 and values for *Δh_ads_* showed good agreement with values from the literature. Measurements on TAM III were evaluated with the TAM Assistant Software™. Measurements on the VP‐ITC were evaluated with Origin®5.0/Origin®7.0. Data was evaluated by manual integration and fitting by PeakFit™. Performance of common fits such as the one binding site model was not possible, because the actual number of binding sites to the resin was not known.

## 3 THEORY

### 3.1 Adsorption isotherm

Adsorption isotherm experimental data used for determination of the equilibrium constant *K*_eq_ is generally fitted using the Langmuir equation:

(1) $$q=q_{\max}\frac{K_{a}C}{1+K_{a}C}$$

where *K*_a_ expresses the protein binding affinity, *C* denotes the protein concentration in the mobile phase under equilibrium conditions, and *q* the protein concentration adsorbed per unit stationary phase. We extrapolate $\frac{q}{C}$ to infinite dilution for the calculation of *K*_eq_:

(2) K eq =lim∂C→0∂q∂C

*K*_eq_ has been used for the calculation of the Gibbs energy change associated with the adsorption of a protein to a stationary phase, Δ*G*_ads_, in numerous publications 29, 30, 31, 32:

(3) ΔG ads =−RTln(K eq )

where *R* is the universal gas constant and T is the temperature.

### 3.2 Isothermal titration calorimetry

An isothermal titration measurement can provide values for the binding enthalpy Δ*H*_ads_, the equilibrium binding constant *K*_eq_, the stoichiometry *n* of the reaction, and consequently the entropy change Δ*S*_ads_, and free energy change Δ*G*_ads_. If the calorimetric measurements are carried out within temperature range, the change in heat capacity at constant pressure, Δ*c*_p_, can also be determined 13.

The enthalpy change associated with the adsorption of a protein to a stationary phase, Δ*H*_ads_, can be determined directly using ITC or in an indirect way using van't Hoff analysis which considers the temperature dependency of the equilibrium constant. In ITC, the heat *Q*, resulting from protein adsorption to a stationary phase, is calculated by integrating the power *P* associated with the interaction of protein and adsorbent over time *t*:

(4) Q ads =∫t1t2P·dt

Q_ads_ is related to the specific enthalpy of adsorption, Δ*h*_ads_ by:

(5) Δh ads =Q ads V·q

V is the volume of stationary phase used.

### 3.3 Heat of dilution

In ITC, the heat of dilution must be accounted for. The most conventional method is to individually subtract (Δ*H*_dil_)^prot^, (Δ*H*_dil_)^gel^, and (Δ*H*_ads_)^ion^ from ΔHads, where (Δ*H*_dil_)^prot^ denotes the heat of dilution of the protein, (Δ*H*_dil_)^gel^ the heat of dilution of the stationary phase, and (Δ*H*_ads_)^ion^ the heat associated with ion adsorption. To determine these heats of dilution for experiments in this study, protein solutions were titrated to the corresponding base matrix lacking the butyl ligand. This way, the heat of dilution could be determined with a single experiment and gave a value that was not influenced by the surface ligand. In other words, we replaced the “classical” way of determining the heat of dilution:

(6) ΔH ads =(ΔH ads ) prot −(ΔH dil ) prot −(ΔH dil ) gel −(ΔH ads ) ion

by applying:

(7) ΔH ads =(ΔH ads ) prot −(ΔH dil ) total

(Δ*H*_dil_)^total^ is the combined sum of (Δ*H*_dil_)^prot^, (Δ*H*_ads_)^ion^, and the heat of dilution of the base matrix, (Δ*H*_dil_)^base matrix^.

It is worth mentioning that the value obtained in this way not only describes the heat of dilution as explained above, but it would also include adsorption enthalpy contributions from any interactions potentially occurring between the sample molecule and the base material. To address this, the value obtained in these blank experiments is described as Δ*H*_blank_ rather than (Δ*H*_dil_)^total^ in Section 4. As long as the resulting value is relatively small compared to the overall Δ*H*_ads_ of the protein and the stationary phase, it can be concluded that these unspecific interactions are negligible. If, however, uncharacteristically large values are observed in these blank experiments, this would be a strong indication that unspecific interactions between the sample proteins and the stationary phase base material might superimpose the actual enthalpies intrinsic to hydrophobic interaction and consequently have a significant influence on the data.

Finally, for calculation of the entropy change associated with the adsorption of a protein to a stationary phase, Δ*S*_ads_, the fundamental property relation for the Gibbs energy is used:

(8) ΔG ads =ΔH ads −TΔS ads

## 4 RESULTS AND DISCUSSION

### 4.1 Equilibrium binding isotherms

**Figure 1:**
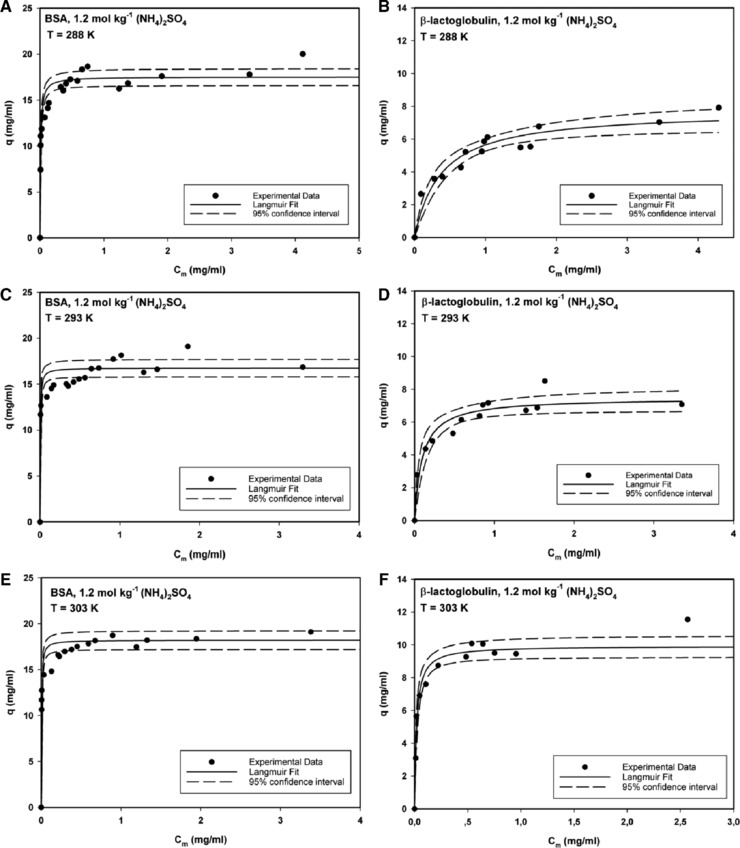
Langmuir isotherms for the adsorption of BSA (A, C, E) and β‐lactoglobulin (B, D, F) to Toyopearl Butyl‐650 M at an ammonium sulfate concentration of 1.2 mol/kg at various temperatures

For many adsorption systems, the Langmuir isotherm model, which assumes adsorption of solutes as a monolayer at equivalent sites without interaction, has been found to be an adequate description. We obtained a series of equilibrium binding isotherms, from which we could extract information on the equilibrium constant *K_eq_* and further apply that value to calculation of the Gibbs free energy, Δ*G*_ads_ for thermodynamic analysis. The amount of protein bound *q* was estimated from the initial slope of the isotherm (Figure 1). The concentration of protein in the stationary phase, *q*, is plotted against the concentration of protein in the liquid phase, *C*_m_. With stationary phase, solely the volume of the beads excluding the void fraction is meant. A careful determination of the initial slope is necessary to quantify the adsorbed protein. For this purpose, protein adsorption can also be fitted with a “physically less justified” model, as discussed in Blaschke et al. 33.

Using the Langmuir equation for describing adsorption in HIC is sometimes questioned, since it is one of the assumptions for this model that no conformational changes occur in the sample molecule.

Yet, Figure 1 illustrates that the only case, where protein adsorption in this study could not be accurately approximated by the Langmuir model was the adsorption of BSA in the intermediate range of protein concentration in the mobile phase. This indicates that additional processes such as unfolding, reorientation, and/or aggregation may simultaneously occur during the protein adsorption, shifting adsorption behavior from mono‐ to multilayer 34. However, for the region investigated by calorimetry at very low mobile phase concentrations, application of the Langmuir fit can be justified. The adsorption behavior of β‐lactoglobulin to Toyopearl Butyl‐650 M at 1.2 mol/kg (NH_4_)_2_SO_4_ concentration could be accurately described with the Langmuir model (Figure 1B, D, and F) over the complete range investigated.

While for BSA, both the shape of the isotherm as well as the observed equilibrium binding capacity remained almost unchanged with increasing temperatures (Figure 1A, C, and E), for β‐lactoglobulin, the isotherm became visibly more rectangular and the observed values for the equilibrium binding capacity increased considerably at higher temperature (Figure 1B, D, and F). This behavior might again be related to the adiabatic compressibility of the proteins. If to a certain extent, conformational changes are necessary to promote more efficient binding to the stationary phase in HIC, than it could be the case, that for a softer protein like BSA, these changes occur already at lower temperatures, while for the more rigid β‐lactoglobulin, higher temperatures are necessary to induce these changes.

### 4.2 Calorimetric measurements

The term Δ*h*_ads_ is used for the specific net molar enthalpy of adsorption determined in the experiments and this term includes the actual heat of adsorption as well as additional contributions. To account for the addition of protein to an excess of stationary phase, we preferred presenting enthalpies as function of added protein instead of protein adsorbed, “*q”*.

We used calorimetric measurements not only to determine the enthalpies of adsorption but to investigate the contributions of unfolding by comparing it with van't Hoff plots. Experiments were conducted with the VP‐ITC calorimeter due to shorter equilibration and spacing times. Due to the fact that ITC measurements of adsorption systems are predominantly carried out as single measurements, it was also of high interest to evaluate the reproducibility of this sensitive method.

Measurements were conducted in the linear range of the isotherm. Protein was titrated into an excess of suspended stationary phase, so that we could assume complete binding of protein. To prevent sedimentation of the beads, a relatively high stirring rate was chosen (see more information in the experimental section). As indicated in the theory section, values for Δ*h*_ads_ were determined only in the Henry's law region of the isotherm (Supporting information Figure S3).

It is known that HIC processes can be driven by enthalpy, entropy, or both, dependent on the salt conditions, stationary phase, and loading 35. For all investigated systems in this study, the adsorption enthalpies were endothermic which indicates an entropically driven process. As expected from calorimetric studies of other HIC systems, the heat flows and the resulting adsorption enthalpies increased substantially with increasing temperature 20, 26, 36.

More detailed information on the experiments as well as depictions of the heat flow in the ITC experiments can be found in the Supporting information.

#### 4.2.1 Effect of base matrix

Dilution experiments were examined according to Eq. (7). Protein was titrated to non‐derivatized base matrix. It resulted in small exothermal enthalpy values within the respective protein‐stationary phase system. For the titration of BSA to the methacrylate‐based Toyopearl matrix HW‐65 at 298 K, average values for Δ*h*_blank_ were –17.0 kJ per mole of protein added at 1.2 mol/kg (NH_4_)_2_SO_4_ and –17.9 kJ/mol at 0.7 mol/kg salt, respectively. For the interaction with β‐lactoglobulin, Δ*h*_blank_ values were less exothermic as presented in Table 1, showing that the interaction is unspecific within the salt system but depending on the protein. This finding is also in accordance with To and Lenhoff's observation that base matrixes “can interact with the protein and affect selectivity and protein recovery” 4.

**Table 1:** Average initial molar enthalpies of adsorption of BSA and β‐lactoglobulin to Toyopearl Butyl‐650 M and enthalpies of dilution to Toyopearl base matrix

		BSA	β‐Lactoglobulin
Salt concentration (mol/kg)	Temperature (K)	Δ*h_dil_* (kJ/mol protein added)	Δ*h_dil_* (kJ/mol protein added)
1.2	298	−17.60	−1.30
0.7	298	−19.38	−1.18
		Δ*h_ads_* (kJ/mol)	Δ*h_ads_* (kJ/mol)
1.2	288	578.83 ± 5.02	128.15 ± 1.81
	293	637.64 ± 15.00	133.64 ± 0.09
	298	708.77 ± 0.55	141.66 ± 3.19
	303	814.91 ± 23.12	180.80 ± 1.85
	308	810.58 ± 14.08	186.03 ± 6.21
0.7	288	133.05 ± 2.00	n.d.
	293	258.61 ± 23.12	89.12 ± 20.92
	298	453.94 ± 31.75	154.71 ± 1.10
	303	658.72 ± 13.16	184.71 ± 1.69
	308	818.95 ± 8.66	199.36 ± 4.10

From these observations we can assume that interactions between protein and base matrix are not significantly influenced by the salt concentration. At the conditions examined in this study, Δ*h*_ads_ is only slightly affected by the dilution enthalpies and the interaction of the protein and the base material. However, for lower salt concentrations and other types of salts and proteins, the influence of the base matrix must be examined in each case.

#### 4.2.2 Effect of temperature

**Figure 2:**
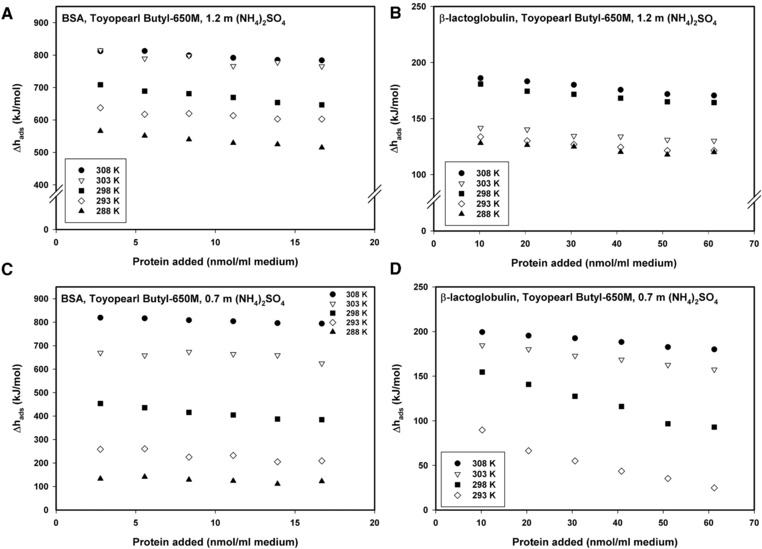
Values of *Δh_ads_* for the adsorption of BSA and β‐lactoglobulin to Toyopearl Butyl‐650 M at an (NH_4_)_2_SO_4_ concentration of 1.2 mol/kg (A and C) and at 0.7 mol/kg (NH_4_)_2_SO_4_ (B and D)

We compared the influence of temperature and salt concentration on the enthalpy change of adsorption as a function of the amount of protein added to Toyopearl Butyl‐650 M (Figure 2). Results are shown for 1.2 mol/kg, promoting complete unfolding, and 0.7 mol/kg, where an intermediate conformational state is assumed (see Supporting information Figure S2B). For BSA, with the exception of studies conducted at 308 K, for which no differences were observed, increasing concentration of salt resulted in higher values of Δ*h*_ads_ (Figure 2A and B) with these differences in Δ*h*_ads_ being more pronounced on lowering the temperature.

Generally speaking, the observed Δ*h*_ads_ values for both proteins changed considerably with the temperature. Given that the isotherm for BSA adsorption showed only very minor changes with increasing temperatures, it seems questionable whether these enthalpy differences can result solely from increased amounts of protein binding to the stationary phase, especially for BSA. It could be assumed that at higher temperatures, conformational changes induced by the adsorption of the protein onto the stationary phase should occur more easily and could here manifest themselves in increasingly positive values for Δ*h*_ads_. It has been hypothesized before, that increased conformational changes could add to the Δ*h*_ads_ of an adsorption reaction due to the energy consumed by the unfolding reaction 26.

**Figure 3:**
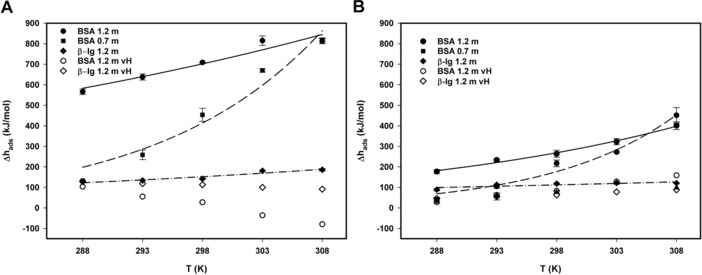
Temperature dependence of the average initial adsorption enthalpies for the interaction of BSA and β‐lactoglobulin. Experimental data was fitted by an exponential function without physical meaning to provide trendlines to guide the eye. Full and open symbols represent calorimetric enthalpies versus van't Hoff enthalpies of BSA and β‐lactoglobulin, respectively. All studies were conducted using the VP‐ITC calorimeter. (A) Toyopearl Butyl‐650 M. (B) Butyl Sepharose 4 FF

Figure 3 summarizes the temperature dependency for both proteins with respect to the average initial enthalpies and compares them to the values obtained by van't Hoff analysis. While the Δ*h*_ads_ determined by van´t Hoff analysis stayed approximately constant or even decreased with temperature, the increase of the calorimetrically measured Δ*h*_ads_ as a function of temperature could be fitted with an exponential function for adsorption to both Toyopearl Butyl‐650 M as well as Butyl Sepharose 4 FF. The exponential increase of Δ*h*_ads_ with temperature at 0.7 molal salt concentration may reflect the exponential increase of protein unfolding as reported by Ueberbacher et al. 7. This strengthens the hypothesis that the calorimetrically measured values of Δ*h*_ads_ include contributions from conformational changes, which are not described by van't Hoff analysis. It may be a result of these contributions that small positive or negative values of the actual adsorption enthalpy, such as reported by Chen and coworkers 17, 20, shift to largely positive values. So far, only differential scanning calorimetry (DSC) data and differences between calorimetric and van't Hoff data provide information about the energetic contribution of conformational changes. Denaturation heats determined by DSC can serve as reference for the magnitude of protein unfolding; however it has to be emphasized not to confuse thermal denaturation with denaturation induced under HIC conditions. Thermal denaturation heats range from 450–550 kJ/mol for globulin‐ and fatty‐acid‐free BSA to approx. 1000 kJ/mol for BSA containing fatty acids in aqueous solution 37. Thermodynamic tables of published heat denaturation data report values around 700 kJ/mol for BSA and from 100–300 kJ/mol for β‐lactoglobulin – depending on the solvent conditions 38. The difference of van't Hoff and calorimetrically determined enthalpies gives values of 550 kJ/mol for 288 K up to approx. 900 kJ/mol at 308 K (Figure 3).

The initial average molar enthalpy change for BSA adsorption accounts for ca. 800 kJ/mol at 308 K and 1.2 mol/kg (NH_4_)_2_SO_4_ (Table 1). For BSA at this salt concentration, a temperature difference of 5 K raises the enthalpy of the adsorption system gradually in steps of 60 to approximately 100 kJ/mol until a plateau is approached at 303 K. For 0.7 mol/kg salt, the steps increase exponentially until the plateau of approximately 800 kJ/mol is reached.

The observed values for Δ*h*_ads_ were much higher for the adsorption to Toyopearl Butyl‐650 M as compared to Butyl Sepharose 4 FF 26, as shown in Figure 3. This observation is consistent with earlier studies by pulse response experiments that found that the temperature dependence as well as the average percentage of protein unfolding was higher for the adsorption to Toyopearl Butyl‐650 M than to Butyl Sepharose 4 FF 7. In this context, it has to be emphasized that this difference might be caused by different ligand densities of the two resins. Since information about the ligand density is only provided by one supplier, the performance can only be evaluated with respect to the resin volume.

For BSA, the minimal differences in the values for Δ*h*_ads_ at 0.7 and 1.2 mol/kg at 308 K (Table 1) reflect the temperature dependence of the unfolding reaction at a salt concentration of 0.7 mol/kg. Ueberbacher et al. estimated nearly 90% unfolding for 0.7 mol/kg salt already at 298 K. Consequently, we can assume that BSA is completely unfolded at 308 K. For the adsorption of β‐lactoglobulin, however, Δ*h*_ads_ values were significantly lower than for BSA (Figure 2 C, D and Figure 3). The same trends were found for the adsorption enthalpies of both proteins to Butyl Sepharose 4 Fast Flow, as previously reported 26. In addition, differences of Δ*h*_ads_ between BSA and β‐lactoglobulin seem to reflect the different extent of unfolding determined by two‐peak evaluation in pulse response experiments and their different isotherm behavior. It has been shown that the (in terms of adiabatic compressibility) “softer” protein BSA unfolded to a significantly higher percentage than the more “rigid” β‐lactoglobulin on Butyl Sepharose 4 FF as well as on Toyopearl Butyl‐650 M 7. By hydrogen exchange experiments, Deitcher et al. observed a sharp transition of the unfolding stability for the adsorption of β‐lactoglobulin B to Butyl Sepharose 4 FF between 0.8 and 1 M ammonium sulfate 39. For the adsorption of β‐lactoglobulin to Toyopearl in this study, large differences between the adsorption enthalpies at 0.7 and 1.2 mol/kg could only be observed at a temperature of 293 K (Figure 2). At all other temperatures, the initial Δ*h*_ads_ values were slightly higher for 0.7 molal salt. This behavior can again be explained by the observation that conformational changes of β‐lactoglobulin are strongly temperature dependent and require higher activation energy 7. A strong temperature dependence for the adsorption of β‐lactoglobulin to another hydrophobic sorbent, Streamline Phenyl, in the presence of various Na_2_SO_4_ concentrations was reported by Bonomo et al. 29.

#### 4.2.3 Effect of surface coverage

As previously observed for the adsorption of BSA and β‐lactoglobulin to Butyl Sepharose 4 FF, values for Δ*h*_ads_ decreased with increasing surface coverage 26. Dias‐Cabral et al. reported the same trend in a flow‐calorimetric study of the adsorption of BSA to PPG‐Sepharose under overloading conditions 36 and attributed this outcome to lower dehydration heats resulting from the decreased amount of water molecules released from the stationary phase because of the increased number of surface sites occupied by proteins. In addition, lateral interactions between the adsorbed proteins manifest as exothermic heats and thus lead to a decrease of Δ*h*_ads_ 35.

In Table 2, thermodynamic quantities are shown for both proteins at 1.2 mol/kg salt concentration. As expected, Δ*g*_ads_ became more negative with increasing temperature for both proteins, showing that the HIC process became more favorable.

**Table 2:** Thermodynamic quantities for the adsorption of BSA and β‐lactoglobulin to Toyopearl Butyl‐650 M at 1.2 mol/kg salt concentration

	Temperature (K)	*K*_eq_	Δ*g*_ads_ (kJ/mol)	Δ*h*_ads_ (kJ/mol)	Δ*s*_ads_ (kJ/mol K)
BSA	288	305.34	−13.70	578.83	2.69
	293	462.78	−14.95	637.64	2.74
	298	461.76	−15.20	708.77	2.43
	303	641.40	−16.28	814.91	2.23
	308	622.47	−16.48	810.58	2.06
ß‐Lactoglobulin	288	24.75	−7.68	128.15	0.47
	293	60.10	−9.98	133.64	0.49
	298	236.65	−13.54	141.66	0.52
	303	346.5	−14.73	180.80	0.65
	308	738.9	−16.91	186.03	0.66

Our future investigations will focus on determining the threshold salt concentration and the temperature for conditions where conformational changes begin to occur. The extensive tailing observed in the studies of the heat flow of adsorption at a specific salt concentration (Supporting Information Figure S2B) suggests the point at which to undertake studies that will define an operating window for the development of process design.

### 4.3 Measurements with two calorimeters

**Figure 4:**
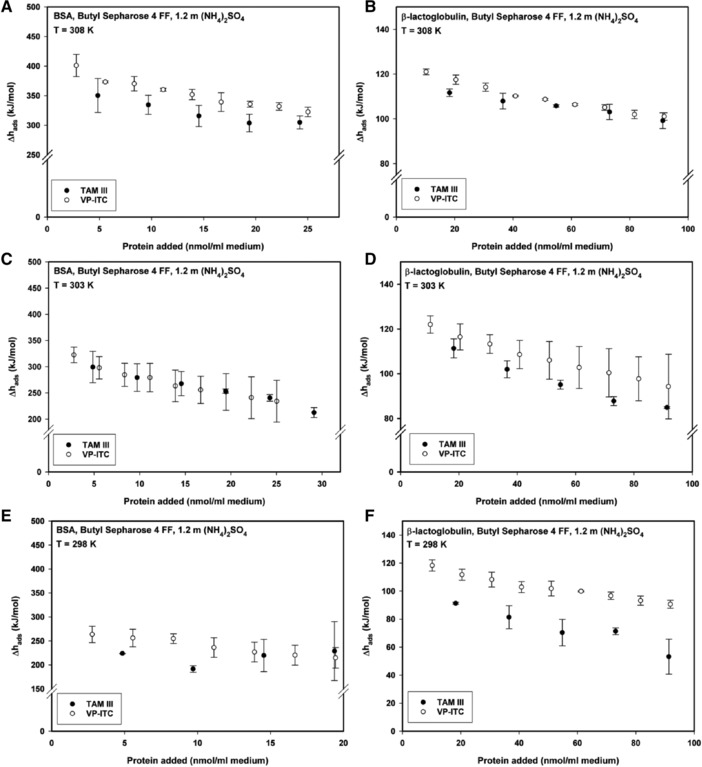
Comparison of *Δh*_ads_ values for the adsorption of BSA and β‐lactoglobulin to Butyl Sepharose 4 FF at 1.2 mol/kg (NH_4_)_2_SO_4_. Adsorption of BSA (A) and β‐lactoglobulin (B) at 308 K. Adsorption of BSA (C) and β‐lactoglobulin (D) at 303 K. Adsorption of BSA (E) and β‐lactoglobulin (F) at 298 K

It is beyond the scope of this study to provide a definite conclusion about which calorimetric system yields more reliable data. However, since literature about ITC in HIC is small and predominantly confined to single measurements, and in awareness of the great sensitivity of calorimeters (baseline SDs in the 20 nW range), we wanted to ensure the data with a second set. This is shown for the HIC systems BSA/β‐lactoglobulin–Butyl Sepharose 4FF. The standard instrument for investigating Δ*h*_ads_ in hydrophobic interaction chromatography has so far been the Thermal Activity Monitor, (TAM) III 17, 18, 19, 20, 40. In the following study, our goal was to determine if intrinsic differences such as cell geometry, cell volume, and stirring mode between two calorimeters (TAM III and VP‐ITC) have an impact on experimental results. Insights into the comparison of various commercially available calorimeters and the involved statistical treatment are given in the previous study 41, 42. For comparison, TAM III data for the measurements at 308 K is taken from 26. The main differences with respect to the calorimetric HIC experiments are listed in Table 3. Figure 4A presents ITC data measured by two calorimeters at different temperatures. The shift of data points along the *x*‐axis is due to the fact that in both cases 1:4 slurries of the stationary phase were used. If the same amount of protein bound/mL gel per injection had been used, a 1:7 slurry would have been required for the VP‐ITC because of the different cell volume of this calorimeter (see Table 3). By using the same ratio of stationary phase to buffer we assured that the protein was titrated to an excess of stationary phase. Consequently, the difference in cell volume led to a different number of injections to account for the same surface coverage at the initial slope of the isotherm. Five injections were performed for the TAM III measurements, while nine injections were used with the VP‐ITC. For example, by considering the amount of added protein per mL gel, injection 4 at TAM III corresponds to injection 7 at the VP‐ITC.

**Table 3:** Selected differences between the TAM III and the VP‐ITC calorimeters

	TAM III	VP‐ITC
Measuring principle	Heat conduction	Differential power compensation
Cell type	Glass ampoules, removable	Hastelloy, fixed in place
Cell geometry/Volume	Cylindrical/1 mL	Coin‐shaped/1.4 mL
Stirring type	Propeller, disposable	Integrated in syringe

At the highest investigated temperature, 308 K, values for Δ*h*_ads_ were in relatively good agreement. For TAM III an average value of 304 ± 15 kJ/mol was determined whereas the Δ*h*_ads_ value for the VP‐ITC was approx 11% higher (336 ± 5 kJ/mol, Figure 4A). A better agreement but also larger SDs were observed at 303 K (Figure 4C). For the adsorption of β‐lactoglobulin to Butyl Sepharose 4 FF at 308 K, average values for Δ*h*_ads_ hardly differ from each other (Figure 4B) whereas, the SDs increase for both calorimeters at 303 K (Figure 4D). For temperatures at 298 K (Figure 4E and F) and lower (data not shown) the comparability decreases. (It is worth noting that values for Δ*h*_ads_ were generally higher for the VP‐ITC measurements.) Generally, comparability of Δ*h*_ads_ values determined with both calorimeters was better at elevated temperatures. We attribute this to the decrease of viscosity with rising temperatures which itself has an influence on the mixing of the adsorbent. Further, longer equilibrating times were required at temperatures of 293 K and below and SDs were higher in these ranges.

## 5 CONCLUDING REMARKS

It is well established that conformational changes are associated with the adsorption of proteins to stationary phases in HIC. Yet, the extent to which these conformational changes take place seems to vary strongly between proteins. As a result, reliable information as to why and how these changes occur, is hard to come by. With this study, we managed to shed some light onto the influence of temperature on the thermodynamic aspects of adsorption of two model proteins in HIC. While in the case of BSA, protein binding itself seems to occur relatively unaffected, increased temperatures do seem to further promote conformational changes in this protein upon adsorption onto a HIC stationary phase surface, which manifest themselves in largely positive Δ*h*_ads_ values. For β‐lactoglobulin on the other hand, the shape of the adsorption isotherm and the resulting equilibrium binding capacity changed considerably at higher temperatures.

While these results indicate that it might be possible to find conditions at which conformational changes during adsorption in HIC do not occur or at least occur to a much lower extent, they also point out once more, that this phenomenon and its various influencing factors such as temperature or salt concentration have to be assessed for each protein individually.

On the other hand, the behavior observed for β‐lactoglobulin, also raises the question whether conformational changes really are an undesirable side‐effect of adsorption in HIC or whether they might even be necessary to some degree to achieve efficient binding.

From a methodological point of view, this study further demonstrates the applicability of calorimetric methods to study the often reversible conformational changes upon adsorption to stationary phases with lower translucency, which cannot be monitored in situ by IR spectroscopy. It is clear that ITC is not a suitable method to directly determine conformational changes in a single experiment. However, enthalpies as functions of salt concentration and temperature can indirectly provide information about the extent of unfolding. In this sense, ITC can serve as an alternative method for “two‐peak” pulse response experiments, with the advantages of lower time consumption (thermostatting) and smaller sample amounts.

With respect to further research efforts in the field, we conclude that when high salt concentrations are required, using lower temperatures might be a way to reduce or altogether avoid conformational changes. Whether such an approach is feasible, will have to be assessed carefully for various different proteins.

## CONFLICT OF INTEREST

The authors have declared no conflict of interest.

## Supporting information

Supplementary material
