# Supplementary material for: Hydrophobic interaction chromatography of proteins: Studies of unfolding upon adsorption by isothermal titration calorimetry
Source: J Sep Sci. 2018 Jun 26;41(15):3069–80. doi: 10.1002/jssc.201800016 (PMC6099299; doi:10.1002/jssc.201800016)
Supplement: Supplementary file 1 — Supplementary material [file JSSC-41-3069-s001.docx]

**Supplementary information**

**Equilibrium binding isotherms:**


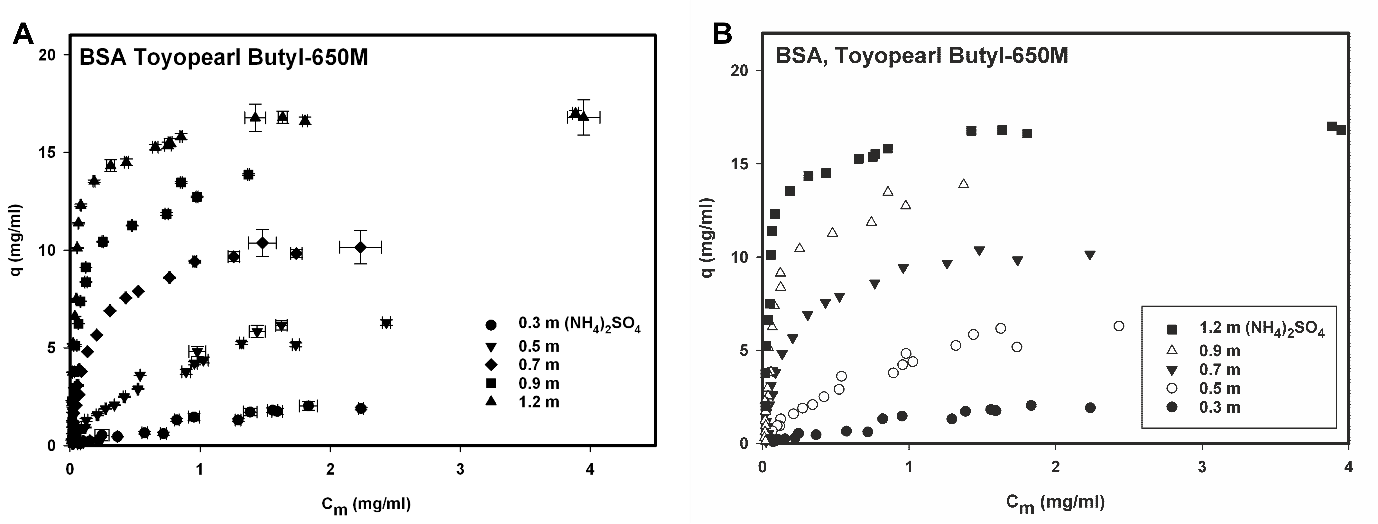


Fig. S1: Adsorption isotherms of BSA to Toyopearl Butyl-650M at different concentrations of (NH_4_)_2_SO_4_ at T=298 K; Standard deviations are represented by bidirectional error bars (A); Average values without error bars to make it easier to distinguish between the individual symbols (B).

Fig. S1 depicts the experimental error of BSA adsorption to the Toyopearl Butyl-650M resin over a range of (NH_4_)_2_SO_4_ concentrations at 298 K, where standard deviations are represented by bidirectional error bars. The experimental error can be attributed to the purity of the model proteins which contain an equilibrium of monomers, dimers and trimers (BSA) and monomers and dimers (β-lactoglobulin), respectively.

For molalities below 0.5, the isotherm shape becomes shallow, showing less favourable adsorption. In this range, it has to be taken into account that the estimated amount of bound protein could falsify the specific adsorption enthalpies.

**Calorimetric measurements:**


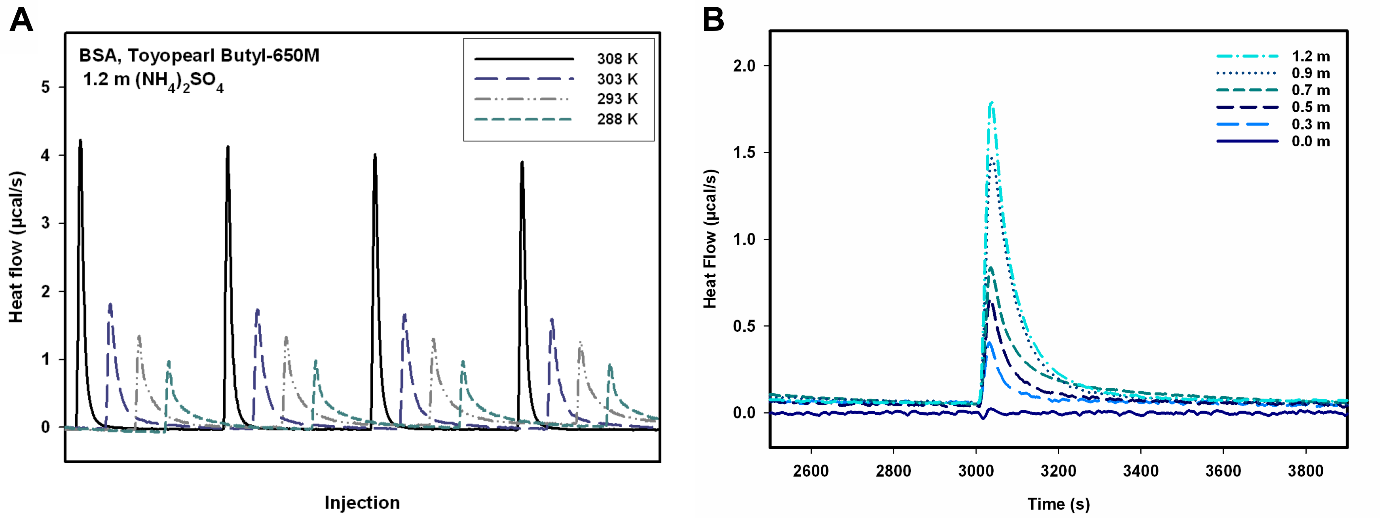


Fig. S2 A: The effect of temperature on the adsorption of BSA to Toyopearl Butyl-650M at an (NH_4_)_2_SO_4_ concentration of 1.2 mol kg^-1^. For more clarity, shifts between the injections are displayed in the graph. B: The effect of (NH_4_)_2_SO_4_ concentration on the adsorption of BSA to Toyopearl Butyl-650M at 298 K. The time scale between the injections is 15 min.

The overlay of heat flow curves in Fig. S2 A illustrates the effect of temperature on the adsorption of BSA to Toyopearl Butyl-650M at 1.2 mol kg^-1^ (NH_4_)_2_SO_4_. As expected from calorimetric studies of other HIC systems, the heat flows and the resulting adsorption enthalpies increased substantially with increasing temperature [20,26,36].


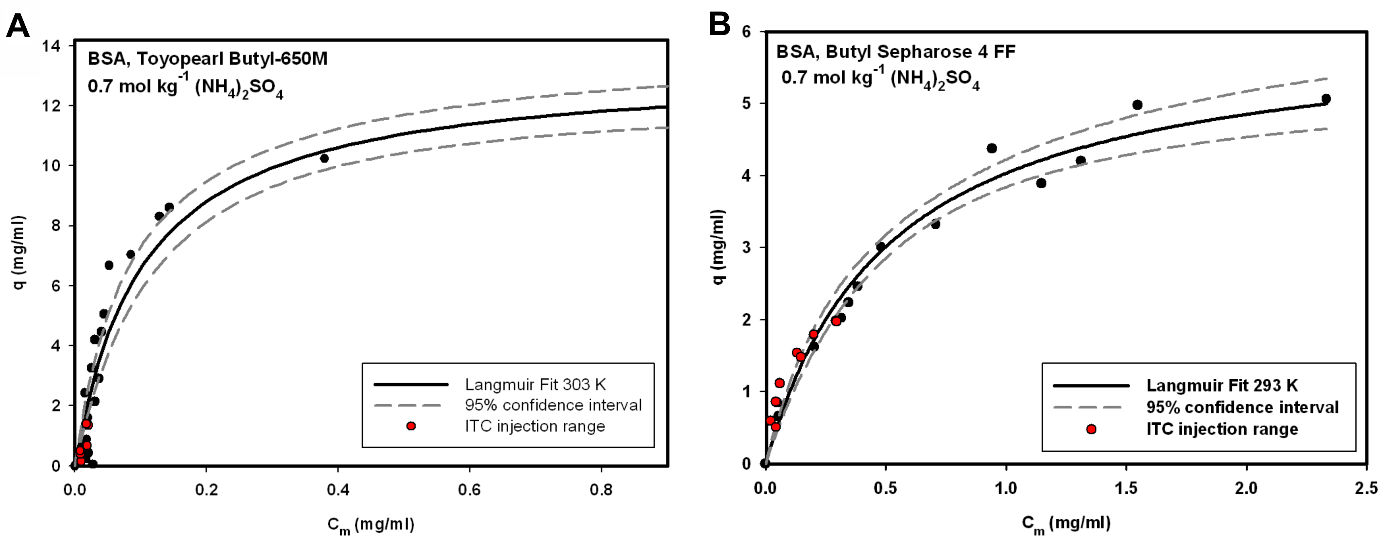


Fig. S3: Isotherms for BSA adsorption to Toyopearl Butyl-650M at 303 K (A) and Butyl Sepharose 4 FF at 293 K (B) at 0.7 mol kg^-1^ (NH_4_)_2_SO_4_ concentration under conditions of the corresponding ITC measurements.

Examples of the isotherm range which was covered by the ITC measurements are shown in Fig. S3. For BSA adsorption to Toyopearl Butyl-650M at 0.7 mol kg^-1^ (NH_4_)_2_SO_4_ at 303 K (Fig. S3 A), an injection containing 1.4 nmol of BSA resulted in a *q*-value from the isotherm of 0.23 mg (1.13 nmol) bound protein per ml gel. The actual amount added in the corresponding ITC experiment was approximately 1 nmol. Therefore, if the whole amount of protein was adsorbed to the stationary phase (V_gel_ = 358 µl) the *q*-value would be 0.17 mg ml^-1^. For a net adsorption enthalpy of 631 µJ, the specific heat of adsorption results in 668 kJ/mol. From the isotherm, we assumed that the largest amount of protein added was bound to the stationary phase. The same was assumed for binding to Butyl Sepharose under these conditions (Fig. S3 B).

### Effect of salt concentration

The heat flows of BSA interacting with Toyopearl Butyl-650M increased with elevated salt concentration, the thermograms corresponding to the isotherm data from Fig S2 are shown in Fig. S3 B. While the areas below the heat flow curves provide quantitative information about the involved heats, the peak shapes indicate kinetic events. The heat flow profile at a salt concentration of 0.7 mol kg^-1^ shows an extensive tailing which represents the longest time period to reach equilibrium again. This might be an indicator for a salt concentration that promotes a shift to a second equilibrium, from the native to the denatured state, resulting in different kinetics [7,32]. Haimer *et al*. showed that at this particular salt concentration and stationary phase most part of the adsorbed protein had been “at least partially unfolded” [10]. Such tailing of the curve was not observed for conditions with 1.2 and 0.9 mol kg^-1^ salt concentration where 100% unfolding is assumed, nor was it observed for 0.3 and 0.5 mol kg^-1^ salt concentration where minor or no unfolding was observed from pulse response experiments [7].
